# Characterization of green synthesized selenium nanoparticles (SeNPs) in two different indigenous halophilic bacteria

**DOI:** 10.1186/s13065-023-01034-w

**Published:** 2023-09-16

**Authors:** Maryam Tabibi, Soheil Aghaei, Mohammad Ali Amoozegar, Razieh Nazari, Mohammad Reza Zolfaghari

**Affiliations:** 1 Department of Microbiology, Qom Branch, Islamic Azad University, Qom, 3749113191 Iran; 2 Extremophiles Lab., Dept. of Microbiology, School of Biology, College of Science, University of Tehran, Tehran, Iran; 3 Department of Microbiology, Faculty of Basic Science, Qom Branch, Islamic Azad University, Qom, Iran

**Keywords:** Biosynthesis, Selenium nanoparticles (SeNPs), Green synthesis, Iran

## Abstract

**Background:**

In the biological method, using nonpathogenic and extremophile bacteria systems are not only safe and highly efficient but also a trump card for synthesizing nanoparticles. *Halomonas elongata QW6 IBRC-M 10,214* (*He10214*) and *Salinicoccus iranensis IBRC-M 10,198* (*Si10198*), indigenous halophilic bacteria, can be used for synthesizing selenium nanoparticles (SeNPs).

**Methods:**

SeNP biosynthesis was optimized in two halophilic bacteria and characterized by UV–Vis, Fourier transform infrared spectroscopy (FTIR), transmission electron microscopy (TEM), field emission scanning electron microscopy (FESEM), X-ray powder diffraction (XRD), zeta potential, and energy dispersive X-ray (EDX).

**Results:**

Optimized conditions for synthesizing SeNPs was at 300 °C at 150 rpm for 72 h and 6 mM or 8 mM concentration of Na_2_SeO_3_. UV–Vis indicated a sharp absorption peak at 294 nm. Spherical-shaped nanoparticles by a diameter of 30–100 nm were observed in FESEM and TEM microscopy images. The produced SeNPs were identified by a peak in FTIR spectra. In XRD analysis, the highest peak diffraction had a relationship with SeNPs. The zeta potential analysis showed SeNP production, and elemental selenium was confirmed by EDX.

**Conclusions:**

Halophilic bacteria, owing to easy manipulation to create optimization conditions and high resistance, could serve as appropriate organisms for the bioproduction of nanoparticles. The biological method, due to effectiveness, flexibility, biocompatibility, and low cost, could be used for the synthesis of reproducible and stable nanoparticles.

**Supplementary Information:**

The online version contains supplementary material available at 10.1186/s13065-023-01034-w.

## Introduction

Nanoparticle synthesis has been interested in recent years. This process is a bridge gap between bulk material and atomic or molecular structure and has much applicability in various fields such as medicine, chemistry, biology, electronics, and energy [1–7]. The importance of nanoparticles depends on their structure and functional characteristics [8].

Selenium is a trace element, sometimes a metalloid, with abundant properties and functions, including semiconductor, thermoelectric and catalytic activities to hydration and oxidation reactions [9, 10]. It acts as an antioxidant and prevents the damage of body tissues against oxidative reactions. Selenium reduces the risk of various cancers, including lung, pancreas, stomach, and intestine cancers and has an inhibitory effect on some bacteria. In spite of many advantages reported, selenium is highly toxic, and its large amounts can have adverse effect. By producing selenium nanoparticles (SeNPs), nanotechnology researchers could reduce the risk of toxicity of this element. The biological activity of SeNPs is highly dependent on their size. Compared to standard selenium compounds, these particles have high biochemical activity so that 5-200 nm of selenium could directly eliminate free radicals in the laboratory environment [11, 12].

Nanoparticle synthesis by physical and chemical methods faces difficulties and impediments that could be managed by the biological approach [13]. The synthesis of SeNPs by this method has been demonstrated to be safe, inexpensive, and eco-friendly, and there is no need for toxic materials [14, 15]. For the bioproduction of metalloid nanoparticles, various microorganisms, such as bacteria, algae, yeast, and fungi, are used [16]. These microorganisms have ability to tolerate a high concentration of toxic heavy metals [17]. The halophilic bacteria can act as bioassay indicators in a polluted environment [18]. The majority of catalytic enzymes in halophilic bacteria play a role in eliminating toxic and chemical materials and also the harmful anions and cations, without leading to the death of these bacteria [19, 20]. As a result, using halophilic bacteria influences both the environment and the survival of organisms [20]. Microorganisms can synthesize nanoparticles by the intracellular and extracellular techniques [14]. In the intracellular method, metal ions penetrate into bacterial cells, and then nanoparticles are produced by the enzymatic reactions, thus reducing agents inside the cell. However, in the extracellular method, the metal ions are put on the cell surface, and nanoparticle synthesis is performed by surface enzymes [21]. While the extracellular method has more advantages, e.g. the recovery facilitation of nanoparticles and low cost, over intracellular approach, many bacteria synthesize nanoparticles by the intracellular method [22]. In other words, the synthesis of SeNPs is carried out by the bioreduction process because reduced metal ion converts to stable biological form [23]. The biosynthesis of SeNPs is assumed to help remove heavy metals from polluted waters around industrial environments. Based on literature review, there are numerous reports on the production of SeNPs by chemical, physical, and the biological method, but scant surveys have examined the biological synthesis of these nanoparticles by halophilic bacteria in Iran. Therefore, this study for the first time biosynthesizes SeNPs using the intracellular method in two halophilic bacteria, *Halomonas elongata IBRC-M 10,214* (*He10214*) and *Salinicoccus iranensis* strain QW6 *IBRC-M 10,198* (*Si10198*), isolated from the native region of Iran. In this light, SeNPs were characterized by the aid of various analytical techniques, comprising ultraviolet-visible (UV–Vis) spectroscopy, Fourier transform infrared spectroscopy (FTIR), X-Ray diffraction (XRD), transmission electron microscopy (TEM), field emission scanning electron microscopy (FESEM), energy dispersive X-ray (EDX), and zeta potential (Additional file 1: Fig. S1).

## Methods

### Chemical substances and bacterial strains

For SeNP biosynthesis, sodium selenite (Na_2_SeO_3_; 172.94 g/mol) was purchased from AppliChem GmbH (Germany). The stock solution was prepared by dissolving Na_2_SeO_3_ (0.345 g) in 10 mL of distilled water, followed by sterilization using a microbiological filter (0.22 μm). *He10214*, a Gram-negative moderately halophilic and heavy metal resistance bacterium, was isolated from Qom Salt Lake (Qom, Iran). Likewise, the moderately halophilic, Gram-positive bacterium, *Si10198*, was isolated from the wastewater of textile industry in Qom [24].

### Culture media and growth conditions

*He10214* and *Si10198* were cultivated aerobically at room temperature in a medium as follows (%, w/v): NaCl, 17.8; MgSO_4_, 0.96; CaCl_2_, 0.036; KC1, 0.2; NaHCO_3_, 0.006; NaBr, 0.0026; MgCl_2_, 0.7; yeast extract, 0.1; glucose, 0.1; proteose-peptone, 0.5; agar, 1.5 in 1 L of water containing 5% (w/v) NaCl, pH 7.2, adjusted with KOH [22].

### Intracellular synthesis of SeNPs by halophilic strains

At first, 1 µL of the inoculum (OD_600_ = 0.1) was transferred to a 100-mL Erlenmeyer flask and treated with different concentrations of Na_2_SeO_3_ (Table 1). Then the production of SeNPs was optimized by incubating cultures in an orbital shaking incubator according to the conditions depicted in Table 1. For the formation of a pellet containing SeNPs and cells, the red culture, showing the formation of SeNPs, was transferred to 50-mL centrifuge tubes, followed by centrifugation at 5000 rpm for 10 min. After supernatant removal, the pellet was resuspended in NaCl (0.9%) and centrifuged (5000 rpm, 10 min); this step was repeated two times. Subsequently, liquid nitrogen was added to the precipitation. Following the formation of a uniform powder, the cell mixture containing SeNPs was ultrasonicated for 5 min. Also, the pellet was centrifuged in Tris-HCL buffer (1.5 M) containing SDS (1%) at 8000 rpm for 10 min (pH 3.8); thereafter, it was washed several times in sterile distilled water. A two-phase (organic-aqueous) system was used to separate SeNPs from cellular debris. For this purpose, following the addition of 2 mL of *n*-octyl alcohol to 4 mL of suspension obtained from the previous stage, the mixture was shaken to admix thoroughly the organic-aqueous phase. The tubes were centrifuged (at 3000 rpm for a period of 5 min) and incubated at 4 °C for 24 h. The organic-aqueous phase was slowly discarded, and the remaining nanoparticles were rinsed with chloroform, ethanol (70%), and distilled water, respectively. Finally, the purified nanoparticle suspension was stored at 4 °C [25]. The suspension was dried in an oven overnight for subsequent analysis.

**Table 1:** Parameters for the optimization of biosynthesis of SeNPs

No.	Parameter	Cond 1	Cond 2	Cond 3	Cond4
1	Na_2_SeO_3_ concentration (M)	2	4	6	8
2	Incubation temperature (^°^C)	30	32	35	37
3	Culturing time (h)	24	48	72	96
4	Shaker (rpm)	150	180	210	240

Cond 1, 2, 3, 4: optimal conditions for SeNPs production

Characterization of SeNPs

### UV–Vis spectrophotometry and FTIR

The first strategy to evaluate the production of nanoparticles was UV–Vis spectroscopy analysis, which was conducted by dispersing the nanoparticle suspension in distilled water. The absorption spectra of the SeNPs were recorded with wavelengths ranging between 200 and 800 nm using Varian Cary 100 UV–Vis instruments (USA). The control curve was obtained from distilled water, and the chemical structure of SeNPs was studied by FTIR analysis. The powder of the samples was mixed with KBr pellets. The FTIR spectrum was recorded in 4395–4495 cm^−1^ and 4388–4488 cm^−1^ regions on a WQF-510 A FTIR spectrometer (Rayleigh, China) at a resolution of 4 cm^−1^ for produced SeNPs using *He10214* and *Si10198*.

### TEM

The size and morphology of nanoparticle powder were determined using TEM. SeNPs synthesized by *He10214* and *Si10198* were characterized by the Zeiss EM900 transmission electron microscope (Germany) at an accelerating voltage of 200 kV.

### FESEM and EDX

For the analysis of the shape of SeNPs, images were taken with SEM (FEI QUANTA 450) at 20 kV HV. EDX was used to ascertain elemental selenium in solid samples. This process was performed by BRUKER Q200 (Germany). X-ray spectrometer analysis and FESEM were simultaneously employed to indicate electron production mapping and point analysis.

### XRD

The crystal structure of SeNPs was characterized by XRD on Ultima IV (Rigaku, Japan) at a voltage of 40 kV with CuKα radiation 1.22 0 A and a scanning rate of 4 degrees per minutes. The crystallite domain size of the synthesized SeNPs was obtained by Debye–Scherrer formula: $\text{D}=\frac{\text{K}*{\uplambda } }{{\upbeta }*\text{cos}{\uptheta }}$.

### Zeta potential

Zeta potential is one of the most important parameters for the stability of nanoparticles in suspension. The zeta potential of SeNPs was analyzed using SZ-100 (Horiba Jobin Yvon SAS, USA). For this purpose, the powder SeNPs were ultrasonicated after dissolving in double distillation water.

## Results

**Fig. 1:**
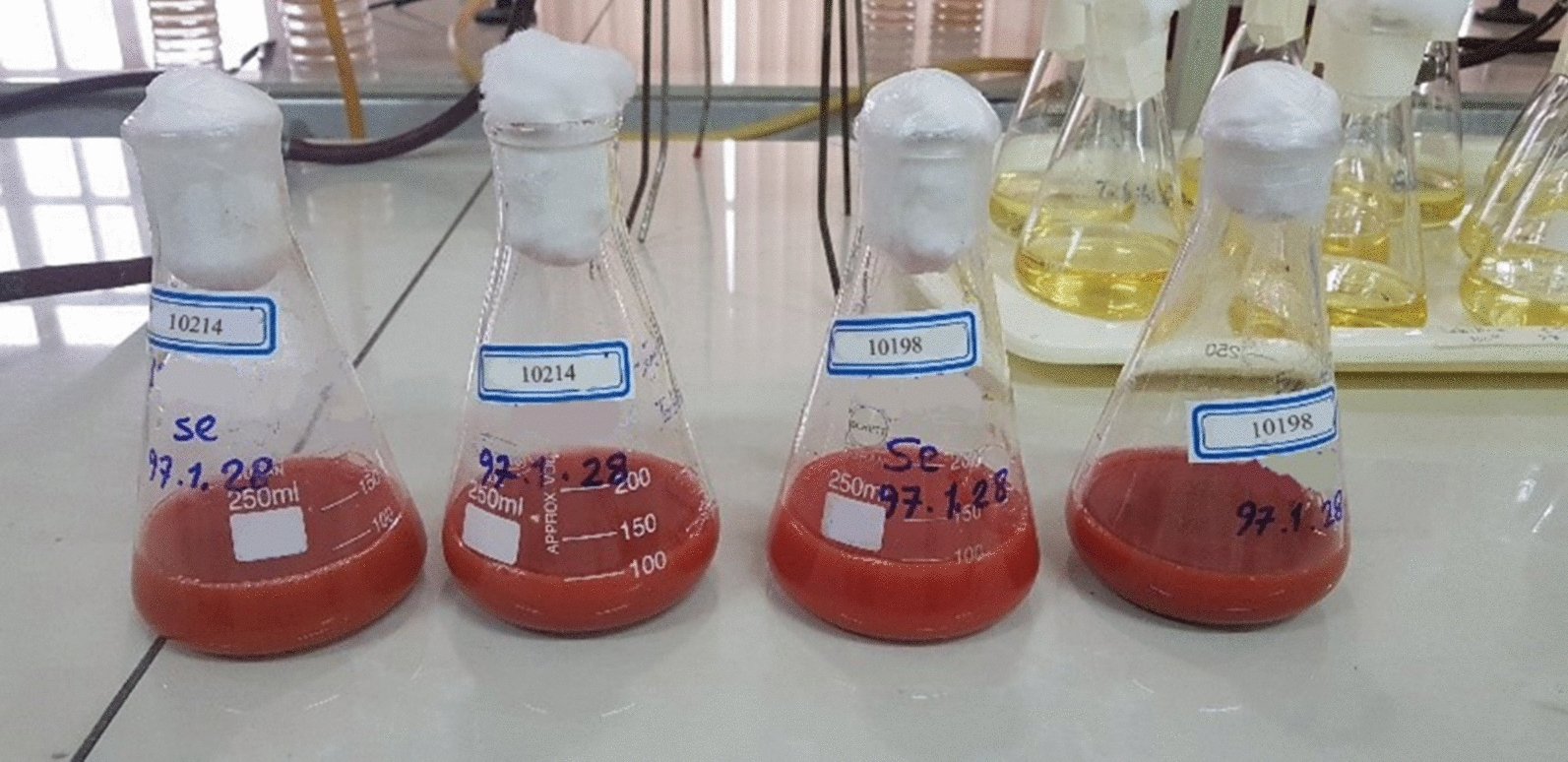
Color change of the culture from light yellow to red, indicating the biosynthesis of SeNPs

*He10214* and *Si10198* as biological factories could synthesize SeNPs successfully. The first evidence in the synthesis of the SeNPs was the medium color change, which became red after the reduction of Se_2_O_3_ to Se_0_ (Fig. 1).

**Fig. 2:**
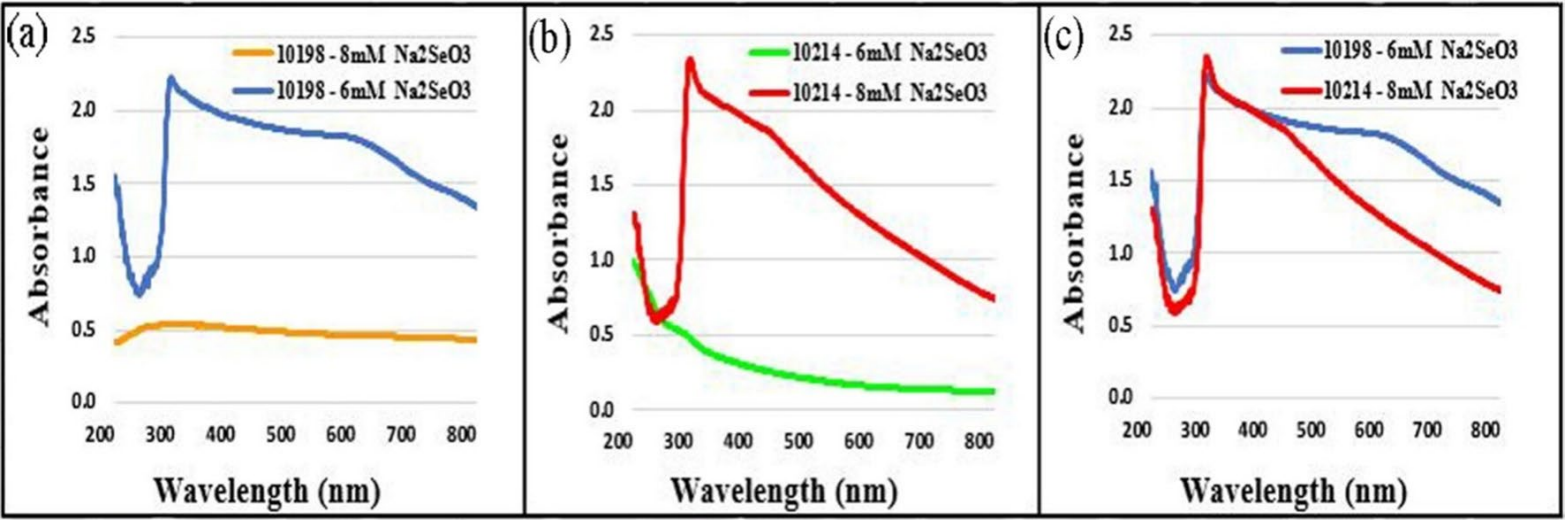
UV–Vis spectroscopy (200–800 nm) of SeNPs biosynthesized by **a** *Si10198* and **b** *He10214* using different concentrations of Na_2_SO_3_. **c** Comparing the highest production rate of SeNPs using *He10214* and *Si10198*

The results from UV–Vis spectroscopy demonstrated the reduction of ${SeO}_{3}^{-2}$ to Se^0^ by the two aforementioned bacteria (Fig. 2). Identification of a strong absorption peak at 294 nm confirmed the formation of SeNPs. Moreover, the maximum production of SeNPs by *Si10198* occurred at 6 mM of Na_2_SeO_3_, and the increasing concentration of Na_2_SeO_3_ did not influence the peak (Fig. 2a). To investigate the yield of SeNPs, the same above-mentioned method was used for *He10214*. The results indicated the maximum production of SeNPs at 8 mM of Na_2_SeO_3_ (Fig. 2b).

**Fig. 3:**
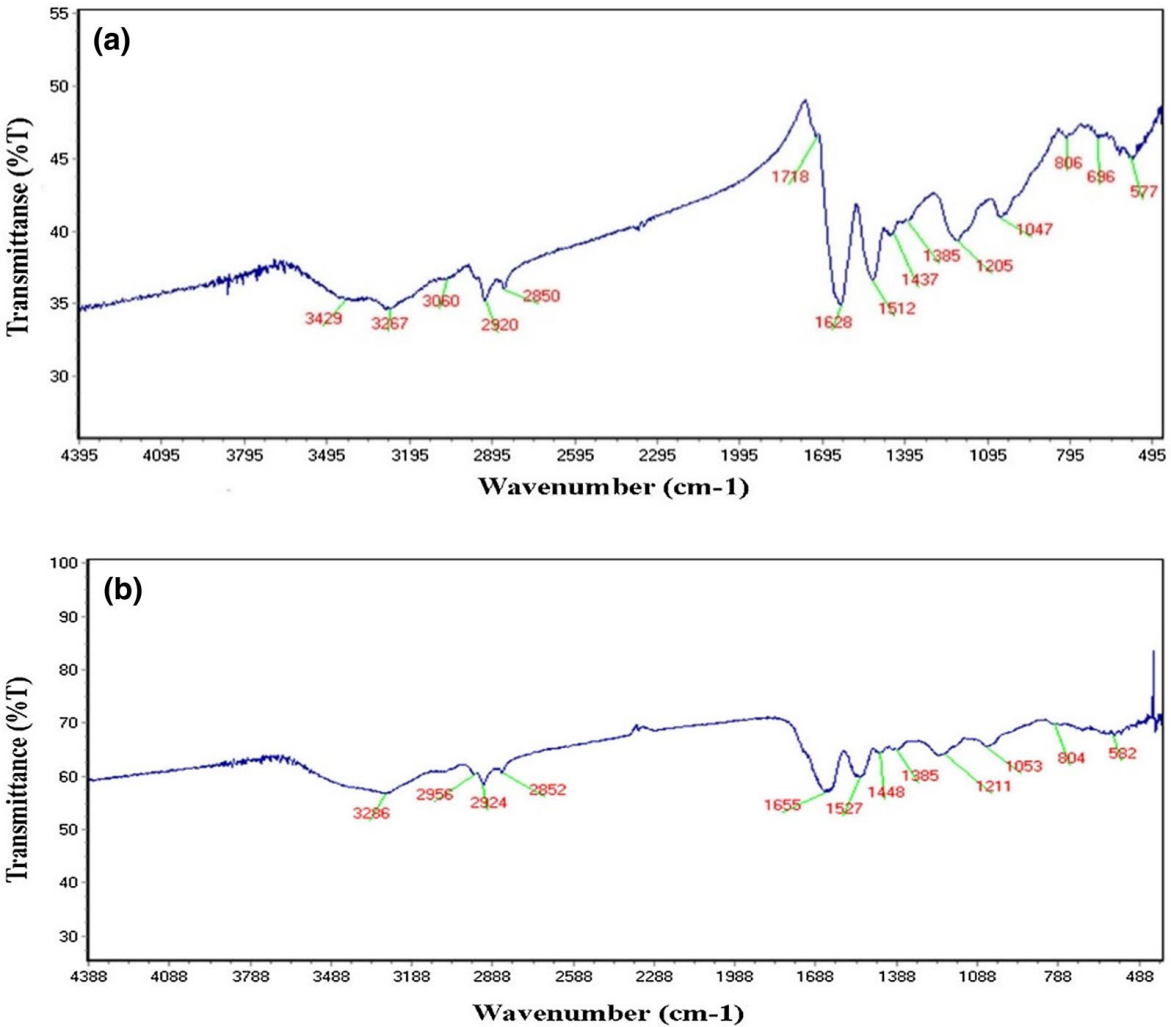
FTIR spectrum of SeNPs biosynthesized by **a** *Si10198* and **b** *He10214*

The chemical structure of SeNPs obtained from *He10214* and *Si10198* was investigated by FTIR. This technique was employed to study the functional groups responsible for the synthesis of SeNPs. The peaks at 2924 cm^−1^ and 2852 cm^−1^ or 2920 cm^−1^ and 2850 cm^−1^ affirmed the presence of ether-methoxy-OCH_3_ groups in *He10214* and *Si10198*, respectively (Fig. 3). The other peaks associated with the functional groups observed in the FTIR spectra of both bacteria are listed in Table 2.

**Table 2:** The bonds of the functional groups of SeNPs biosynthesized by *Si10198* and *He10214*

Functional group	*Si10198* Wavelength (nm)	*He10214* Wavelength (nm)
Amide A (in protein)	3268	3267
C–H in –CH_3_	2956	3060
C–H in > CH_2_	2924	2920
C–H in > CH_2_	2852	2850
C=O	1655	1628
Amide I (in protein)	1527	1512
Amide II (in protein)	1448	1437
–CH_2_/–CH_3_	1385	1385
COO^–^	1211	1205
Amide III	1053	1047
C–O, C–C, C–O–H, C–O–C	804	806
Phosphoryl group	582	577

**Fig. 4:**
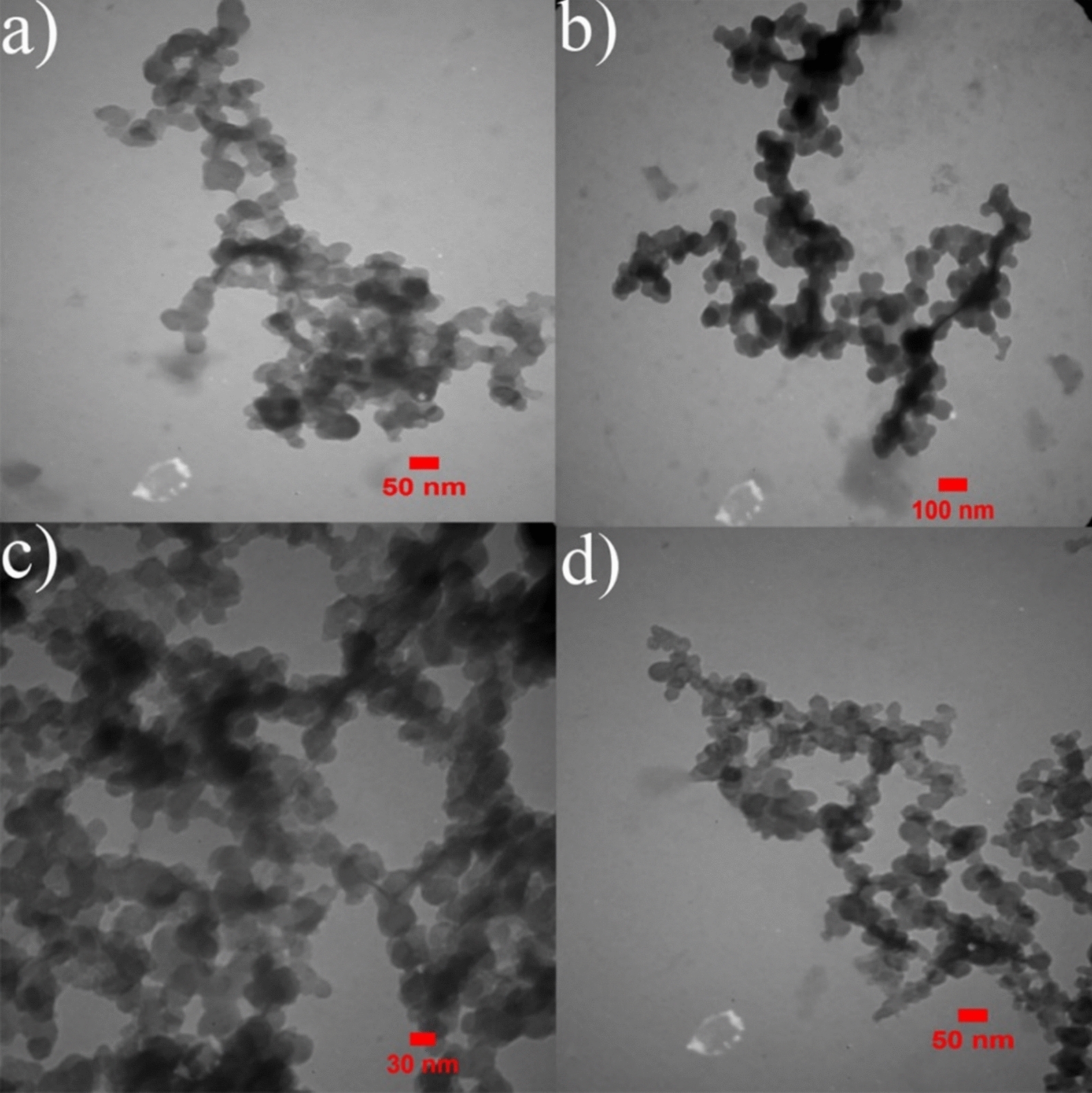
TEM image of SeNPs biosynthesized by *Si10198* (**a** and **b**; 6 mM) and *He10214* (**c** and **d**; 8 mM)

**Fig. 5:**
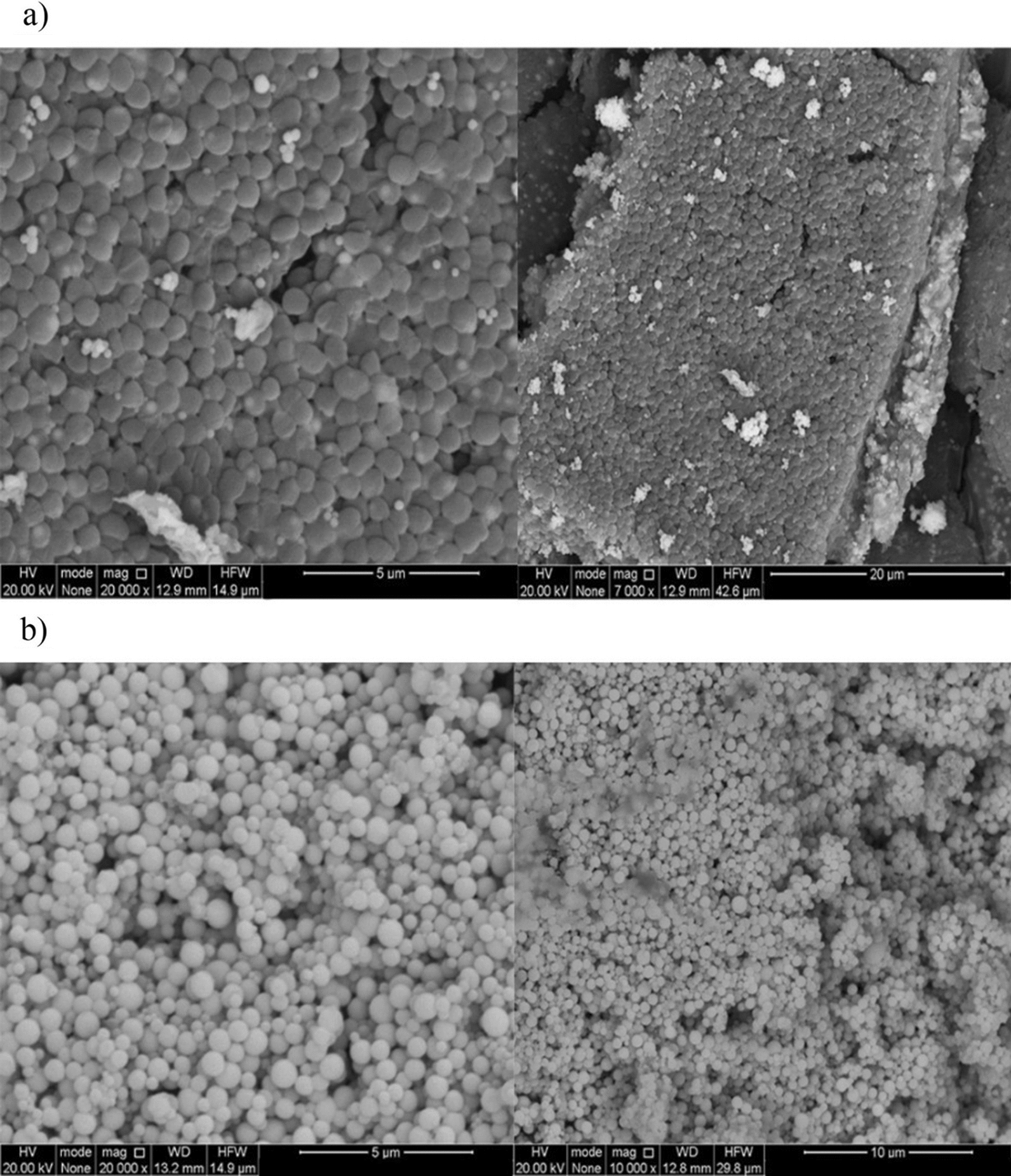
FESEM image of SeNPs biosynthesized by **a** *Si10198* and **b** *He10214*

The electron microscopic images of SeNPs is represented in Fig. 4 and shows a spherical shape with a mean diameter of around 50–100 nm and 30–100 nm in 6 and 8 mM of Na_2_SeO_3_, synthesized by *He10214* and *Si10198*, respectively. Images obtained from FESEM confirmed the spherical shape of SeNPs biosynthesized by the two bacteria (Fig. 5).

**Fig. 6:**
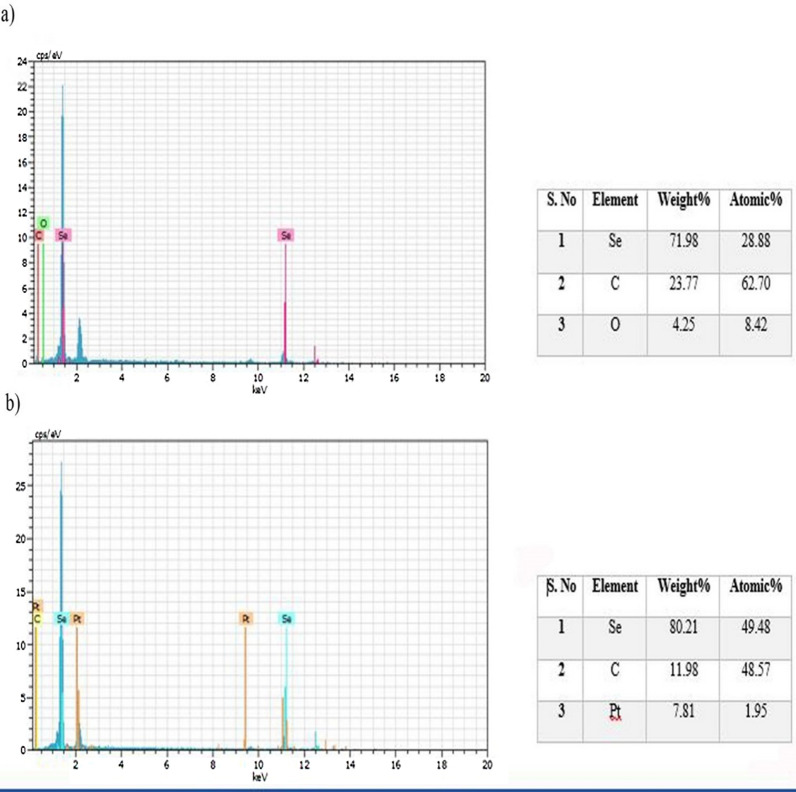
EDX analysis confirming selenium element in **a** *Si10198* and **b** *He10214*

**Fig. 7:**
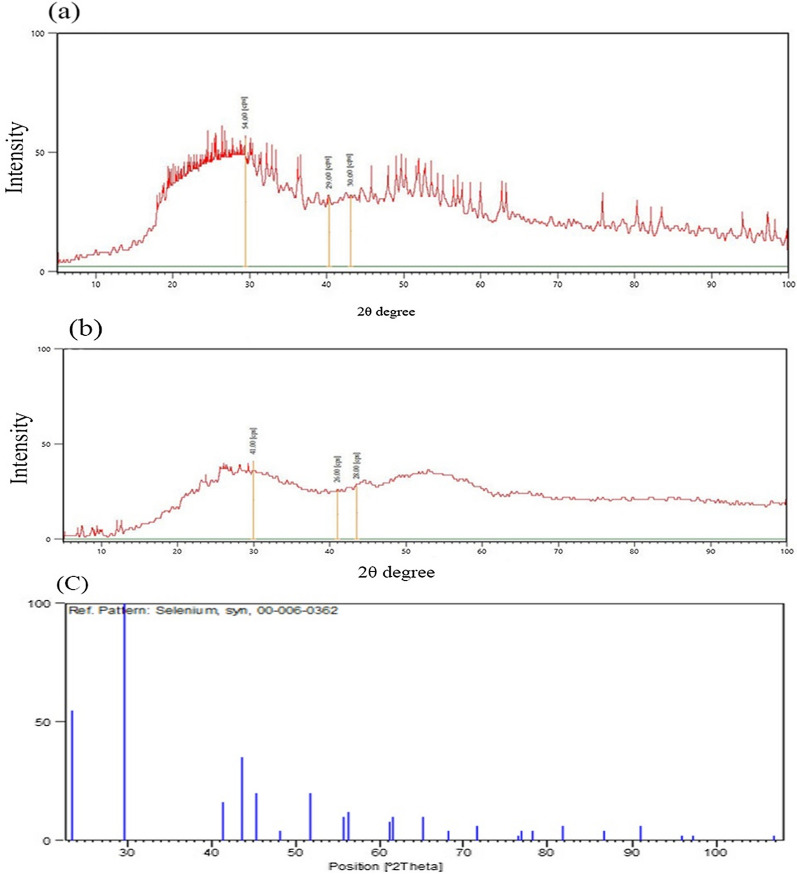
X-ray analysis confirming the presence of SeNPs in **a** *Si10198* and **b** *He10214*, and **c** SeNP standard pattern on XRD

**Fig. 8:**
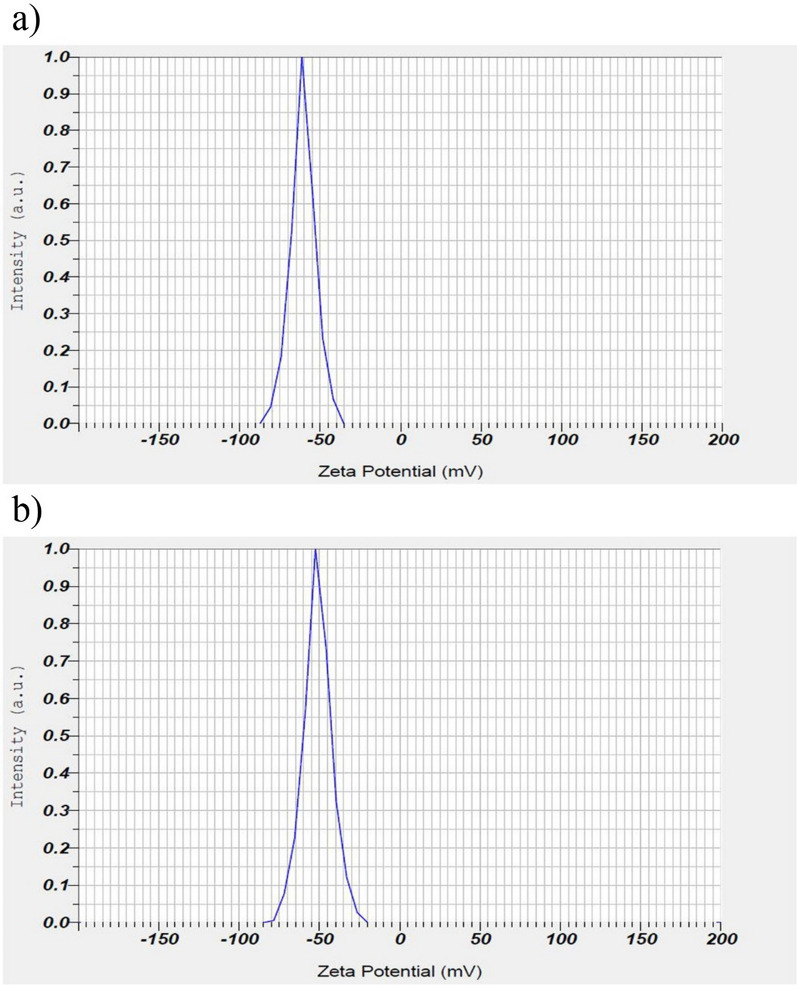
Zeta potential measurement of the SeNPs using **a** *Si10198* and **b** *He10214*

The EDX analysis of SeNPs synthesized by *He10214* and *Si10198*, as shown in Fig. 6, exhibited that absorption peaks in both bacteria were 1.4 (SeLα), 11.7 (SeKα), and 12.5 KeV (SeKβ). XRD analysis also confirmed the synthesis of SeNPs by the two bacteria (Fig. 7). The highest intense peak was detected at 54 cps, and the diffraction peak was about 29.5 (2θ). Owing to the impurity of nanoparticles and adherence of bacteria, the peak in our study was lower than other studies [12, 26], and the standard diffraction peak at 2θ was observed at ~ 30, with the intensity of 101. The zeta potential of the synthesized SeNPs by *Si10198* was found to be − 60.6 mV (Fig. 8a), while that of *He10214* was − 51.2 mV (Fig. 8b).

## Discussion

The present study explored that *He10214* and *Si10198* as a biological factory could successfully synthesize SeNPs. The high compatibility of these bacteria in specific environmental conditions and their structural characteristics shows that halophilic bacteria have a prominent role in the production of SeNPs. While the high amount of selenium is toxic for humans, plants, and microorganisms, owing to the antioxidant and antimicrobial features, this element has a leading function in the protection of the ecosystem. As a result, SeNP biosynthesis in optimized conditions using bacteria resistant to heavy metals and harsh circumstances would be cost-effective. In this survey, SeNP production was optimized by 6 or 8 mM of Na_2_SeO_3_ for *He10214* and *Si10198*, respectively at 150 rpm, 30 °C for 72 h [15]. The red color of the culture affirmed the presence of SeNPs, as indicated in several studies [4, 10, 15, 27].

### UV–Vis analysis

In the present study, the UV–Vis spectroscopy results exhibited that ${SeO}_{3}^{-2}$ decreased to Se^0^ by both *He10214* and *Si10198*, which is consistent with a finding achieved in the Srivastava et al.’s [26] study, in which a peak at 300 nm was obtained from the synthesized SeNPs using some species of Lactobacillus. Overall, the observation of the peak between 200 and 300 nm indicates the presence of SeNPs [9, 28–32]. Based on the UV–Vis spectra of biosynthesized SeNPs by both bacteria in different concentrations of Na_2_SeO_3_ (6 mM and 8 mM), *He10214* could tolerate the higher concentration of selenium compared to *Si10198*. Thus, it seems that *He10214* outperform *Si10198* in refining the selenium-rich wastewater of factories.

### FTIR analysis

The same as fingerprints in humans, FTIR can be used to identify nanoparticles. This work compared the FTIR patterns and matched all the adsorbents in the two molecules spectrum. The peaks indicated the existence of a biopolymer associated with the SeNPs, which is found in cell walls as a reducing factor for the synthesis of SeNPs [27, 31]. Fritea et al. [32] found an association between the absorption peak of 2918 cm^−1^ and SeNPs, which probably implies the presence of a biopolymer in the cell wall. On the other hand, *He10214* is Gram-negative and *Si10198* Gram-positive bacteria; the differences in the type and thickness of their cell wall have caused slight variations between the absorption pattern of these two bacteria.

### TEM analysis

The morphology of SeNPs by TEM revealed that these nanoparticles had a spherical shape with a mean diameter of about 50–100 nm and 30–100 nm in 6 mM and 8 mM Na_2_SeO_3_, which were synthesized by *He10214* and *Si10198*, respectively. According to previous studies, the spherical shape of the produced nanoparticles confirms the presence of SeNPs [30, 33]. While these results are comparable with a study in which the size of these nanoparticles was observed at 50–150 nm [34], the size of the biosynthesized nanoparticles varies depending on the host producing these particles and also the conditions of production. The sizes of biosynthesized SeNPs obtained by TEM was in the range of 20–80 nm [26]. In this regard, Avendaño et al. [33] and Forootanfar et al. [35] have observed SeNPs in the sizes 100–500 nm and 80–220 nm, respectively.

### FESEM analysis

An electron microscope FESEM is more suitable for imaging nanoparticles due to providing high resolution at a low voltage. As reported in numerous studies, the spherical and uniform shape of synthesized SeNPs is observable through the FESEM technique [36].

### EDX analysis

The EDX analysis determines the concentrations of the constituent elements and their composition by scanning via an electron microscope [37–43]. Quantitative analysis of SeNPs synthesized by *He10214* and *Si10198*, as demonstrated in Fig. 6, reflected that absorption peaks in *He10214* and *Si10198* bacteria were 1.4 (SeLα), 11.7 (SeKα), and 12.5 KeV (SeKβ). The highest peaks were related to the selenium element, and their weights in *He10214* and *Si10198* bacteria were 71.98% and 80.21%, respectively. The presence of carbon (C), oxygen (O), and platin (Pt) elements in EDX analysis might be due to capping with proteins and cell membranes, reactions of reduction, and also the stability of produced SeNPs. Potassium element was identified in the *He10214* strain and the oxygen element in the *Si10198* strain, which is likely due to different types of cell walls. In multiple investigations, such as Fernández-Llamosas et al. [10], Srivastava et al. [26], Cremonini et al. [44], Sharma et al. [12], and Dhanjal et al. [45], the selenium peak was explored at 1.4 (SeLα), which is the highest peak related to selenium synthesis.

### XRD analysis

XRD analysis confirmed the synthesis of SeNPs by *He10214* and *Si10198* bacteria. The highest intensity peak was 54 cps, and the diffraction peak was about 29.5 (2θ), which supports the study conducted by Alagesan and Venugopal [46]. Because of nanoparticles impurity and bacteria adherence in the current study, the peak was lower than that in previous studies [26, 27, 47], and standard diffraction peak at 2θ was observed at ~ 30, with the intensity of 101. In a surface absorption study related to particle morphology and suspension stability, zeta potential analysis is practical [48].

### Zeta potential analysis

The zeta potential of the synthesized SeNPs suspended was found to be − 60.6 mV by *Si10198* and − 51.2 mV by *He10214*. These results of our study are in conformity with the findings of Mollania et al.’s [49] investigation in which the zeta potential reflected a high negative charge on the SeNPs (− 46.86 mV). However, in previous studies, the zeta potential of this nanoparticle has been reported in different rates; for instance, Srivastava and Mukhopadhyay [30] observed the zeta potential of SeNPs in − 7.7 mV, and Vekariya et al. [50] reported a zeta potential of − 28.8 mV. Also, the zeta potential value for fabricated SeNPs using fungi was reported as − 22.9 mV by Zare et al. [51]. Capping and stabilization of nanoparticles by bacterial proteins cause a high negative charge on the SeNPs. Stabilized particles are not transformed to black amorphous form when were kept for a long time [34, 45]. Since the zeta potential of SeNPs synthesized by Si10198 was more negative; therefore, this strain had a higher effective role in stabilizing SeNPs. Research is being completed to produce, evaluate and apply nanoparticles, and human knowledge in this regard is increasing day by day. One of the limitations of nanoparticle production is storage and particle size maintenance. After nanoparticle synthesis, an important matter is the synthesis of nanoparticles that are more stable and remain in the nanoscale for a longer period, or in other words, the accumulation of nanostructures is prevented. The protection of body balance has been indicated to be dependent on the normal function of antioxidant compounds and enzymes [52]. The generation of oxidative stress has a relationship with the altered function of the electron transport chain in the inner membrane of mitochondria [53]. The growth of biologic pollution, particularly certain drugs, heavy metals, and radiation, can eliminate the body’s hemostasis and increase the free radical production. Oxidative stress development results in destructing not only body cells but also biological molecules [52].

## Conclusions

In the present study, SeNPs were synthesized in a bacterial system using the biological method, which is safe, effective, eco-friendly, inexpensive, and flexible. The findings of this survey suggest that halophilic bacteria *He10214* and *Si10198*, found in the native region of Iran, have capability of producing nanoparticles. Under optimal conditions, the two bacteria could produce SeNPs with 100 − 30 nm. These nanoparticles, in virtue of biocompatibility and high potential, could be used as antioxidant, anticancer, and antimicrobial agents in future studies.

### Supplementary Information

**Additional file 1: Fig. 1.** Synthesis of SeNPs using bacteria.

## Data Availability

The datasets used and/or analyzed during the current study are available from the corresponding author on reasonable request.

## Abbreviations

EDX: Energy dispersive X-ray
FESEM: Field emission scanning electron microscopy
FTIR: Fourier transform infrared spectroscopy
Na_2_SeO_3_: Sodium selenite
SENPS: Selenium nanoparticles
TEM: Transmission electron microscopy
UV–Vis: Ultraviolet–visible
XRD: X-ray diffraction
