# Supplementary figures and images for: Characterization of green synthesized selenium nanoparticles (SeNPs) in two different indigenous halophilic bacteria
Source: BMC Chem. 2023 Sep 16;17(1):115. doi: 10.1186/s13065-023-01034-w (PMC10504756; doi:10.1186/s13065-023-01034-w)

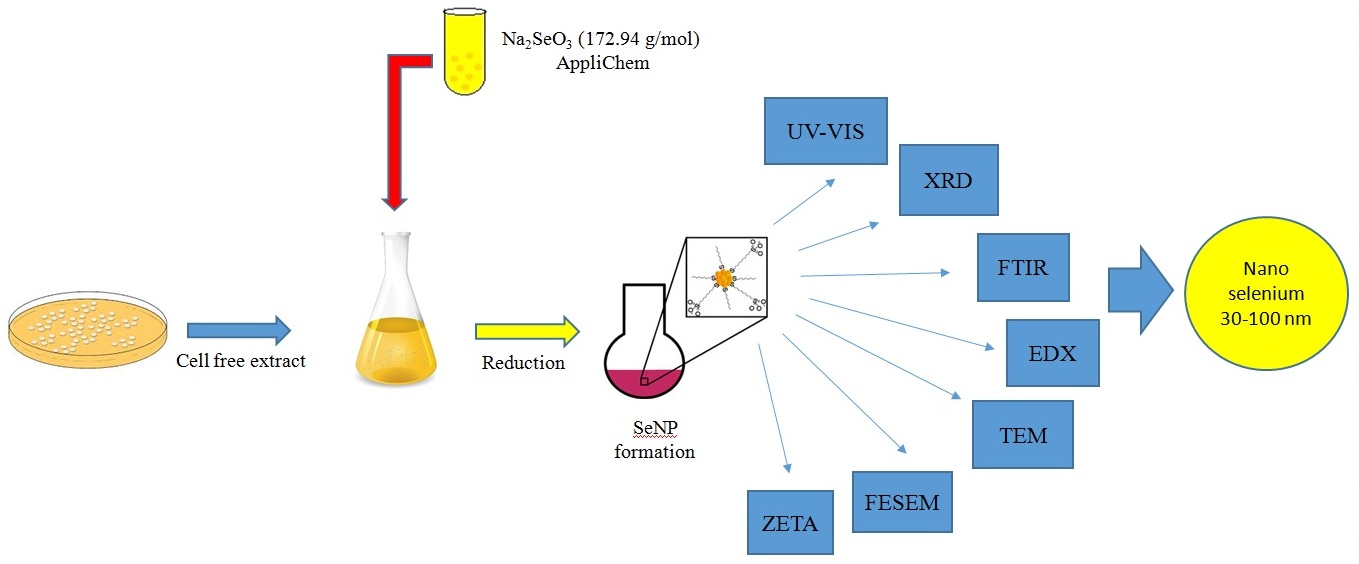


**Fig. S1.** Synthesis of SeNPs using bacteria.

Supplement: Supplementary file 1 — Additional file 1: Fig. 1. Synthesis of SeNPs using bacteria. [file 13065_2023_1034_MOESM1_ESM.docx]
